# Fleeting victories: COVID-19 and the temporary improvements in asthma care behaviours

**DOI:** 10.1016/j.lanepe.2024.100957

**Published:** 2024-06-11

**Authors:** Rama Vancheeswaran, Meera Mehta

**Affiliations:** a West Herts NHS Teaching Trust, Vicarage Road, Watford, WD180HB, UK; b Imperial College Healthcare NHS Trust, St Mary's Hospital, Praed Street, W2 1NY, UK

Global studies of respiratory diseases, particularly asthma, noted a decrease in exacerbations of up to 63%^1^^,^^2^ during the COVID-19 pandemic. This unexpected reduction provided crucial respite in hospital admissions, helping to meet the increased demand on healthcare resources posed by the pandemic. It is hypothesised that the decrease in asthma exacerbations (Fig. 1: data from the Office for National Statistics, UK) was due to a decrease in the circulating common respiratory viruses, further supported by patient shielding.^2^ While evidence suggests other contributing factors, such as improved adherence to medications leading to fewer patient self-reported exacerbations,^3^ a detailed analysis of known patient modifiable risk factors has been lacking.

**Fig. 1:**
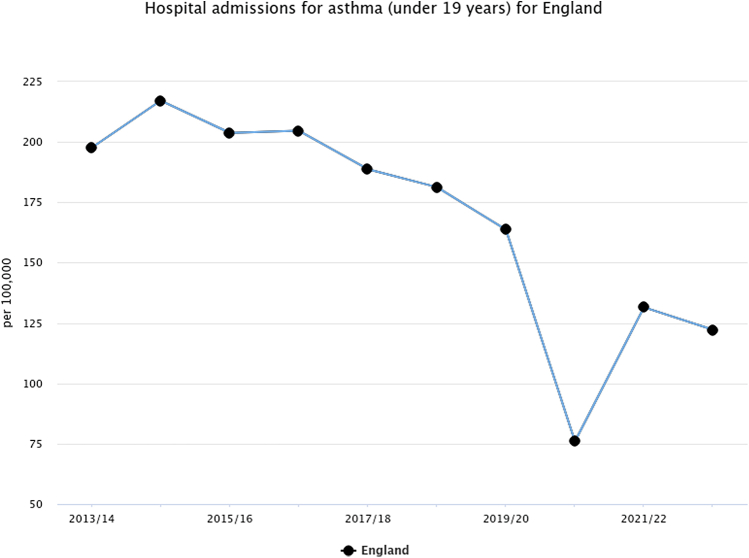
Hospital Admissions for asthma (under 19 years) for England.

Source: OHID, based on NHS England and Office for National Statistics data.

In this issue of *the Lancet Regional Health-Europe*, Mukherjee et al.^4^ present a timely challenge to the prevailing view with a compelling, large-scale, retrospective, observational analysis of several modifiable risk factors. Using data from a large UK primary care database, the authors utilised a sophisticated multilevel generalised linear mixed model to analyse over 2 million asthma patients over four years: pre-pandemic (2019) and during the pandemic (2020–2022). The database included available risk factors such as smoking status, inhaled corticosteroid prescriptions, prophylactic influenza and pneumococcal vaccinations, asthma annual reviews and self-management plans. Outcomes included respiratory tract infections and asthma exacerbations, measured by health care utilisation and prednisolone prescriptions.

Like other studies, the authors observed a transient reduction in asthma exacerbations during the pandemic compared to the pre-pandemic year of 2019 (67.8% in 2019, 52.4% in 2020, 55.4% in 2021, 62.5% in 2022). This trend was evident in GP recorded events, prednisolone prescriptions, emergency department visits, and hospitalisations. Importantly, the study highlights significant modifiable risk factors particularly patient behaviours, that were associated with improved asthma outcomes. While causality is not established, better asthma outcomes were linked to patient self-reliance and empowerment. There was a notable rise in the adoption of asthma self-management plans (+31.9% in 2020, +66.3% in 2021, +99.3% in 2022), higher rates of influenza vaccination (+245% in 2021 and +284% in 2022), improved inhaled corticosteroid compliance (+2.9% in 2020, +2.1% in 2022) and decrease in active smokers (−3.7% 2020, −3.8% 2021 and −2.7% in 2022).

The reduction in asthma exacerbations was not linked to increased asthma annual reviews (−4.9% in 2020, -1.9% in 2022) further supporting the notion that patient behavioural changes rather than increased engagement with healthcare professionals led to better outcomes. Interestingly, there was no increase in pneumococcal vaccination uptake, suggesting that reduced vaccine hesitancy was specific to viral infections and did not extend to all pathogens. The study confirmed a reduction in all respiratory infections, most marked in 2020, which gradually diminished by 2022 (URTI: −46.2% 2020, −51.6% 2021, −27.9% in 2022, LRTI -50% 2020, −46.7% 2021, and −22.8% 2022).

Asthma exacerbations have nearly returned to the pre-pandemic levels seen in 2019.^5^^,^^6^ Several studies have identified modifiable risk factors for reducing asthma exacerbations including viral infections, ongoing exposure to allergens, non-adherence with medication, smoking, obesity and co-existing conditions such as reflux disease and rhinitis.^7^ However, psychosocial interventions targeting these factors have seen limited success.^8^ Mukherjee et al. made a commendable effort by incorporating some patient and public opinion in interpreting the study results. This could have been enriched further by concurrently capturing patient knowledge, attitudes towards risk, and behaviour triggers during the pandemic.

The authors correctly recommend simple measures, such as mask use to improve asthma control, and briefly comment on the positive behavioural changes. However, while there is some recall of the pandemic, there is an urgent need for timely research using the comprehensive behavioural toolkit to provide insights into sustaining the positive changes observed during COVID-19.

The well-established link between higher deprivation levels and asthma is based on factors such as pollution, poor housing, smoking, poverty, reduced education, mistrust in authorities and misinformation. These factors often coincide with a disregard for protective health measures.^9^^,^^10^ While this study records deprivation, it does not explore the association with health inequality. It would be valuable to determine whether asthma behaviours varied based on perceptions of COVID-19 risk across different social and ethnic groups. Surveying how COVID-19 risk is communicated to diverse demographic groups and identifying which groups change behaviour could allow for more targeted and effective health communication strategies. Such insights are crucial for health care policy, particularly in detecting and addressing disparities.

Mukherjee et al. provide a comprehensive primary care database study on pandemic-associated modifiable risk factors, offering a valuable foundation for future investigation. These insights could enhance preparedness and healthcare planning for future pandemics.

## Contributors

RV-Conceptualisation and writing: original draft, review and editing, MM-writing: review and editing.

## Declaration of interests

The authors have no competing interests.
